# Modular, Discrete Micromixer Elements Fabricated by 3D Printing

**DOI:** 10.3390/mi8050137

**Published:** 2017-04-26

**Authors:** Krisna C. Bhargava, Roya Ermagan, Bryant Thompson, Andrew Friedman, Noah Malmstadt

**Affiliations:** 1 Mork Family Department of Chemical Engineering and Materials Science, University of Southern California, Los Angeles, CA 90089, USA; kcbhar@gmail.com (K.C.B.); ermagan@usc.edu (R.E.); acfriedm@usc.edu (A.F.); 2 ReoLab Inc., San Diego, CA 92121, USA; bryantth@usc.edu; 3 Department of Biomedical Engineering, University of Southern California, Los Angeles, CA 90089, USA

**Keywords:** 3D printing, microfluidics, micromixing

## Abstract

3D printing facilitates the straightforward construction of microchannels with complex three-dimensional architectures. Here, we demonstrate 3D-printed modular mixing components that operate on the basis of splitting and recombining fluid streams to decrease interstream diffusion length. These are compared to helical mixers that operate on the principle of chaotic advection.

## 1. Introduction

Mixing of miscible fluids in micro- and millimeter scale channels is difficult to accomplish without agitation of flows with integrated actuators. Flows are strictly laminar at typical flow rates, characterized by Reynolds Numbers several orders of magnitude below the transition region (2000–4000) [1]. Consequently, convection has little influence on co-flowing liquids, leaving diffusion as the dominant mechanism by which streams mix. This can be quite slow in microfluidic systems for biochemical processing, where fluid reagents often contain high-molecular-weight compounds with low diffusion coefficients [2]. These reagents are also often rare or costly, and reducing their consumption is imperative for the success of research programs. This poses a problem for engineers, who must balance the reagent volume resident to their microfluidic system whilst achieving sufficiently high mixing efficiencies. Therefore, methods of improving mixing efficiencies must shorten the diffusion distance of mixture components through clever architecting of channel geometries or partitioning of fluids into compartments such as droplets [3,4]. The literature broadly explores two device architectural mechanisms by which this may be accomplished: lamination and chaotic advection [5]. Briefly, lamination involves arranging inlet co-flows of differing reagents in alternating lanes across the channel, accomplished either by constructing interdigitated inlets to a single mixing channel or by splitting co-flows and reassembling them repeatedly [6,7,8,9]. This method is relatively straightforward, reducing the characteristic diffusion distance by a predictable amount. Mixing co-flows with chaotic advection involves stretching and folding flow lines through engineered channel geometries or making channels with sufficiently three-dimensional architectures [2,10,11,12]. This is somewhat less straightforward, with performance enhancements to mixing seen at higher Reynolds Numbers than is typical to many applications in biochemical processing. Both lamination and chaotic advection ultimately require the construction of multi-layer channel devices with three-dimensional features, a significant challenge for microfabrication technologies. Previously, we introduced a system of discrete elements for assembling three-dimensional microfluidic circuits with integrated process sensors [13,14,15,16]. We leveraged additive manufacturing techniques (i.e., “3D printing”) to achieve device designs with sophisticated interior architectures and create a system of interconnects based on self-aligned interference fits. These devices are significantly less difficult to manufacture than traditionally micro-machined, multilayer monolithic lab-chips, and enable engineers to construct systems that are suitable for mass manufacturing. Each element in our growing library is designed to enable the use of lumped-parameter modeling and network analysis techniques familiar to discrete element-based electronics design and adjusted for the nuances brought on by mass-manufacturing of systems in which multiple fluid reagents may be used. However, the range of channel sizes achievable through additive manufacturing is limited as compared to traditionally micro-machined devices, motivating the development of mixers with a wide coverage of resident volumes and hydraulic resistances. In this report, we introduce a new class of discrete elements engineered for high-efficiency mixing across a range of flow rates based on the principle of lamination. The intricate three-dimensional architecture of these elements is made possible through additive manufacturing. We compare the mixing performance of these lamination mixers to that of helical mixers that operate via chaotic advection. We also introduce a new method of experimentally quantifying mixing efficiency and characterize mixer performance with respect to key engineering design trade-offs between mixing efficiency, resident volume, and hydraulic resistance.

## 2. Results and Discussion

### 2.1. Design of Laminator Discrete Elements

**Figure 1:**
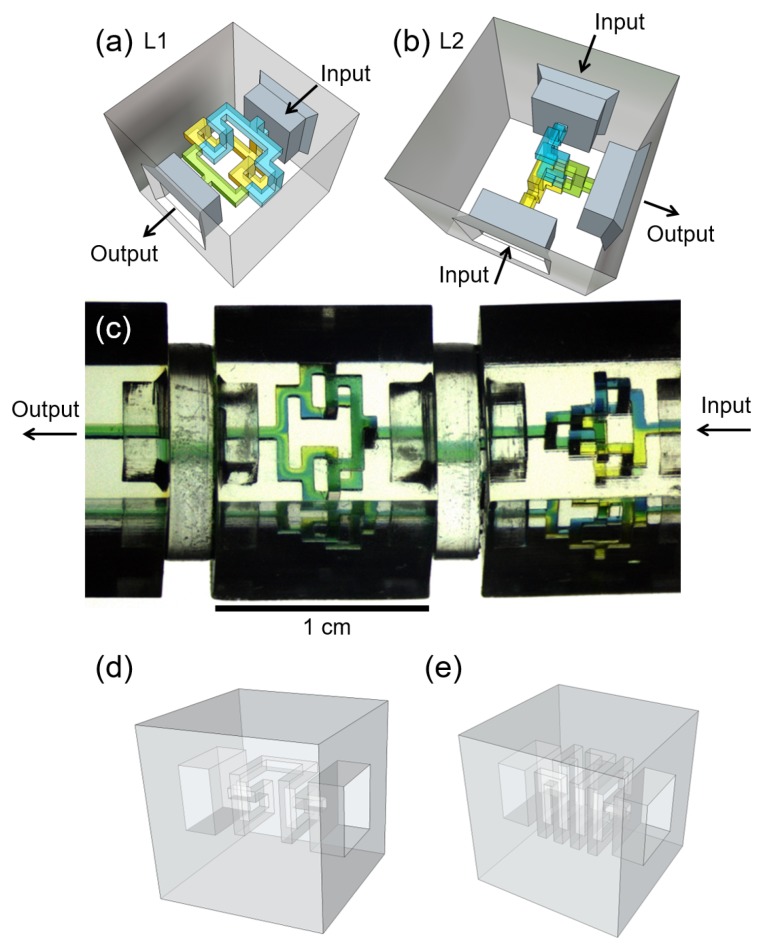
Computer-aided drafting (CAD) representation of the L1 (**a**) and L2 (**b**) laminator discrete elements introduced in this report. The inlet reagent flow paths are coloured blue and yellow, and the outlet flow is coloured green. More views of these laminator elements are presented in Figure S1. Top-view photograph of the second L1 device (with 90${}^{\circ }$ rotation with respect to the first L1), within an L1 + L1 series configuration, showing the doubling of two lamellae to four, and then eight aiming for mixing enhancement. (**c**) Flow is from the right to left; each block is 1 cm long on each side. CAD representations of the helical 5G (**d**) and helical 10G (**e**) devices studied in this report and compared to laminator devices.

Two variations of our laminator discrete element were designed and manufactured using stereolithography [13,14,15,16], as seen in Figure 1. In the first device, dubbed “L1” throughout this report (Figure 1a), a co-flow of two laminated miscible fluid streams is introduced into the element from a single inlet, split into separate channels such that each fluid stream is isolated, split again individually to duplicate each isolated flow, and merged in the outlet channel in an interdigitated fashion. This results in alternating layers of the two fluids. This same basic procedure is performed in the second device, dubbed “L2” (Figure 1b), where instead flows of differing fluids are directly introduced into the element from two inlets. In this manner, both components divide the diffusion distance in half from the inlet(s) to the outlet. The internal microfluidic circuit of each element is designed such that the hydraulic resistance from any inlet to the outlet of the element is always the same. This is important to guarantee that each channel segment fills at the same rate, minimizing the risk of gas bubbles being formed in areas of the network where there are closed loops. This is also particularly important for the L1 device, in which the inlet co-flow is assumed to be 50% filled with either fluid. Both devices rotate the relative arrangement of inlet flows by 90${}^{\circ }$ in the plane perpendicular to the direction of flow, as seen in Figure 1c. Thus, in placing the element in a network, one must consider how inlet fluids are managed both before entering and after exiting a laminator element. For example, in the L2 element, reagents are directly introduced into the component in one plane, but exit the component stacked on top of one another, perpendicular to the plane of their introduction. In the L1 element, reagents must enter the component in a co-flow with the interface between them in one plane, but exit such that this interface is perpendicular to its original orientation. In other words, if the L1 component is not oriented correctly, co-flows will simply be re-arranged into the same configuration they were already in, but in a plane perpendicular to their inlet arrangement. These elements can be arranged in series with one another to continually shorten the diffusion distance and enable geometric enhancement to the mixing performance of the overall system. This is seen the L1 + L1 system shown in Figure 1c. Note that the second component has been rotated 90${}^{\circ }$ with respect to the first component, such that the inlet co-flow is split correctly into two isolating channel segments internally and functions to enhance mixing. To gauge the mixing efficiency of the L1 and L2 laminator devices within the context of reagent volume and network resistance, we compared the mixing efficiency to elements previously described in [13]. Figure 1d,e shows two of these helical devices, dubbed “H 5G” and “H 10G”, differentiated by their internal path length, number of turns, and consequent hydraulic resistance.

In order to assess the L1 and L2 laminator devices within the context of these parameters, we compared their mixing efficiencies to those of three more elements previously constructed in our laboratory [13]. These components were designed with convenient hydraulic resistance (to pure water) values of 1, 2.5, 5, and 10 GPa-s-m3 (“SP 1G”, “S 2.5G”, “H 5G”, and “H 10G” respectively, where “SP” stands for “straight-pass”, “S” for “snake”, and “H” for “helix”). The helical devices are shown in Figure 1d,e; all devices are shown in Table S1.

### 2.2. Quantifying Mixing Efficiency

Stereolithography enables the facile routing of microfluidic channels in three dimensions, but has much larger manufacturing tolerance than traditional micromachining. For example, in the processes used to construct devices presented in this report, tolerances can be as high as 30 μm, whereas micro CNC tolerances are often <5 μm and semiconductor processing tolerances are submicron [14]. Variation in channel sizes in discrete microfluidic elements directly propagates fluid handling performance errors, implying the need for simple and versatile empirical device characterization methods. For example, in the laminator devices presented in this report, manufacturing imprecision may cause the internal inlet-to-outlet distribution of hydraulic resistances to differ from their intended symmetry. In addition, the internal microfluidic network is sufficiently three-dimensional that chaotic advection effects may cause unexpected variation in performance, especially at higher flow rates. Combined with variable interfaces between reagent co-flows and reagents and channel walls, these effects motivated us to develop a method of rapidly quantifying the flow rate-dependent mixing efficiency of discrete microfluidic elements.

**Figure 2:**
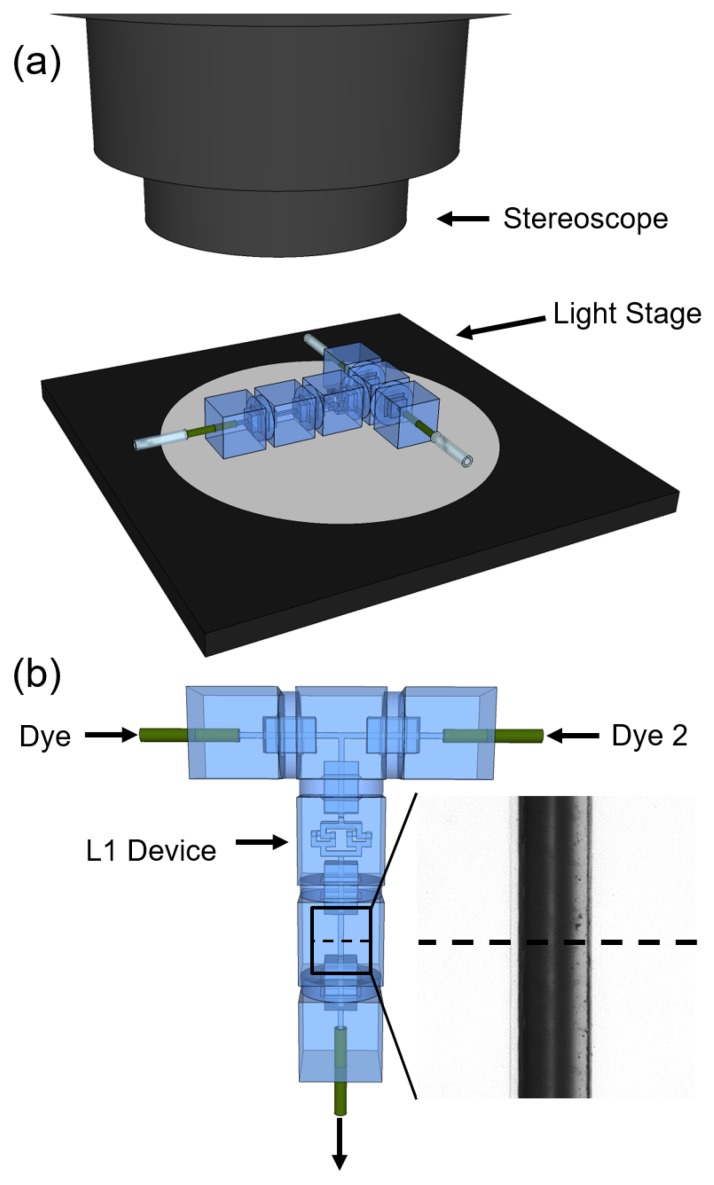
(**a**) CAD representation of the experimental setup used to determine mixing efficiency. The systems were placed on a light stage to illuminate the straight pass channel where the microscope was focused to take images (inset); (**b**) signal intensity (such as in Figure S2) was measured down a line crossing the channel perpendicular to the direction of flow and used to determine mixing efficiency with in-house developed algorithms.

Figure 2 describes the experimental setup used to measure mixing performance. The library of discrete microfluidic elements used in this report is tabulated in Table S1, including terminal characteristics to flow, internal network representation, and nomenclature used throughout this report for each element. Elements were assembled so that two inlet reagent flows are merged to form a co-flow, and then pass through the laminator device or combination of devices being characterized. This is followed by a simple straight pass element (“SP 1G”) that is inspected with a stereoscope and high resolution camera such that the interface between differing reagent co-flows is perpendicular to the imaging plane. A water stream containing a dye of known diffusivity and high absorbance was flowed through one inlet, merging with a flow of pure water from the other inlet. A dual-syringe, single driver syringe pump was used to manage inlet flows such that flow rates were well-matched to one another and the dyed stream would be mixed with water in a 1:1 ratio. The total inlet flow rate was then varied across a range of typical laboratory values and a monochrome image was captured for each flow rate, indicating the extent to which diffusive mixing had occurred.

An image processing algorithm was developed to quantify the flow rate-dependent mixing performance of the devices measured (Figure S2). At a given flow rate, the intensity profile of the channel cross-section was measured at the centre of the observed SP 1G component and averaged. The resultant data was then normalized to a scale in which the intensity of the pure water was set to 1 and the intensity of pure dye was set to 0. An edge finding algorithm was used to find the interior of the channel and extract the intensity data interior to the channel. A line was fit to the data corresponding to a perfectly mixed scenario, taken at an extremely low flow rate. This line was subtracted from remaining data corresponding to relevant flow rates to correct for uneven illumination within the channel (more example data from before and after this correction is found in Figure S3).

Mixing efficiency was determined by measuring the average absolute deviation (AAD) from the mean for each processed intensity frame, or AAD, expressed as:

(1) $$AAD\left(Q\right)=\sum_{n=1}^{N}\frac{|I_{n}\left(Q\right)-\langle I\left(Q\right)\rangle |}{N}$$

Here, $I_{n}$ represents a processed intensity data point along the measurement line and indexed by pixel number *n* as a function of the flow rate, *Q*. *N* represents the total number of pixels in the set, or interior of the channel. 〈I〉 represents the mean intensity of that line. In other words, the AAD quantifies how poorly reagents are mixed in the channel by assessing how much the intensity distribution in the channel deviates from a mean-valued, flat line. To compute a measure of efficiency, a condition for perfect non-mixing was used to establish the upper theoretical limit of AAD. We assumed this was 0.5, or the AAD of a channel in which exactly one half contains dye and the other half is translucent and in which diffusion is not possible. The mixing efficiency μ was therefore calculated as:

(2) $$\mu \left(Q\right)=1-\frac{AAD(Q)}{0.5}$$

### 2.3. Device Performance and Engineering Trade-Offs

**Figure 3:**
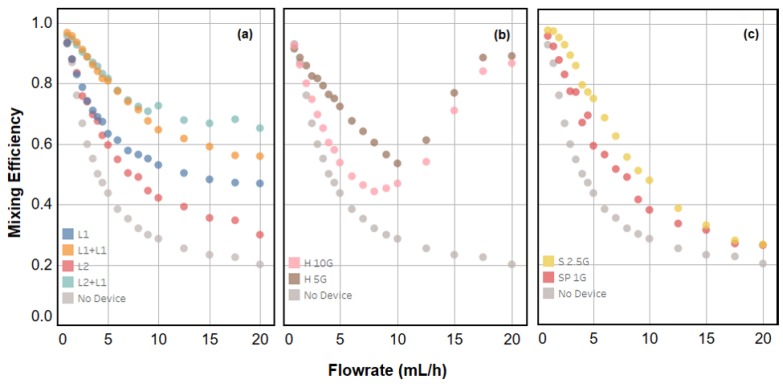
The flow rate-dependent mixing efficiency for all devices and configurations studied, including (**a**) the new laminator designs, (**b**) helical elements (**c**) and planar channel elements previously introduced in [2]. Device nomenclature is given in Table S1.

Figure 3 shows the flow rate-dependent mixing efficiency for all devices and configurations characterized in this study. A standard T-junction element with no subsequent mixing elements was measured for comparative purposes (data labelled “No Device”). Figure 1a shows the results for laminator devices and series configurations of laminator devices. As expected, the efficiency declines as flow rate increases. The L1 and L2 devices and their series combinations show significant improvement in efficiency over the T-junction over a large range of flow rates, as well as nearly linear, comparable performance to one another at low flow rates. Like the T-junction, the efficiency for each individual laminator device appears to plateau at a minimum value with increasing flow rate. This is consistent with the expectation that faster flow rates will result in less diffusion and, hence, clear separation of dyed and translucent fluid lamellae. None of the systems approach an asymptotic efficiency of zero at high flow rate; this is due to the fact that some Taylor dispersion-mediated mixing occurs [1]. All laminator systems plateau at a higher mixing efficiency than the “no device” system. These systems both have a longer path length than the “no device” case and some three-dimensional turns that would be expected to result in some mixing by chaotic advection. The specific value upon which the L1 and L2 devices converge appears to deviate from one another despite the intended design of both devices resulting in the same number of lamellae. We suggest that this is a direct result of imperfect manufacturing. For example, the L1 device outperforms the L2 device because of the added channel structure at the inlet which acts to split the flows: imperfect splitting leads to pre-mixing of fluids before lamination is accomplished. This effect may also cause disparities in the behaviour between L1 + L1 and L2 + L1 series configurations, with the latter resulting in better mixing above 5 mL/h. However, any series combination of devices certainly leads to a significant gain in mixing performance over individual devices. This is consistent with the notion that each device essentially doubles the number.

In designing discrete element microfluidics, two hydraulic terminal characteristics are of importance to designers: resistance to flow [13,14,17] and resident volume. The inherently parallel arrangement of the microfluidic network within both L1 and L2 elements implies that their hydraulic resistance is of minimum consequence to most networks of interest. However, the increase in total channel length relative to other elements in our library implies that the resident volume of each component can also significantly impact a designer’s reagent volume budget. Broadly speaking, a designer seeks to minimize the addition of resident volume to their microfluidic network design while maximizing mixing efficiency at the flow rate of operation. The resistance of the device will possibly affect this flow rate depending on whether a constant flow rate or constant pressure driven flow is being utilized, but can be managed in conjunction with other resistive components in the network.

In order to assess the L1 and L2 laminator devices within the context of these parameters, we compared their mixing efficiencies to those of three more elements previously constructed in our laboratory [13]. These components were designed with convenient hydraulic resistance (to pure water) values of 1, 2.5, 5, and 10 GPa-s-m3 (“SP 1G”, “S 2.5G”, “H 5G”, and “H 10G” respectively, where “SP” stands for “straight-pass”, “S” for “snake”, and “H” for “helix”). The helical devices are shown in Figure 1d,e; all devices are shown in Table S1. These devices use the same channel size as others described here, but achieve increased hydraulic resistance by routing a single channel in a snake or helical shape with square walls. The benefit of helical structures to mixing is two-fold: at low flow rates, the longer channel length allows for increased diffusion times and therefore enhanced mixing efficiencies, while at high flow rates mixing is enhanced due to chaotic advection [1]. This is directly evident in Figure 3b: the flow rate-dependent efficiency of both devices have minima around 10 mL/h. By comparison, Figure 3c shows devices with no three-dimensional structure that do not benefit from chaotic advection at higher flow rates.

**Figure 4:**
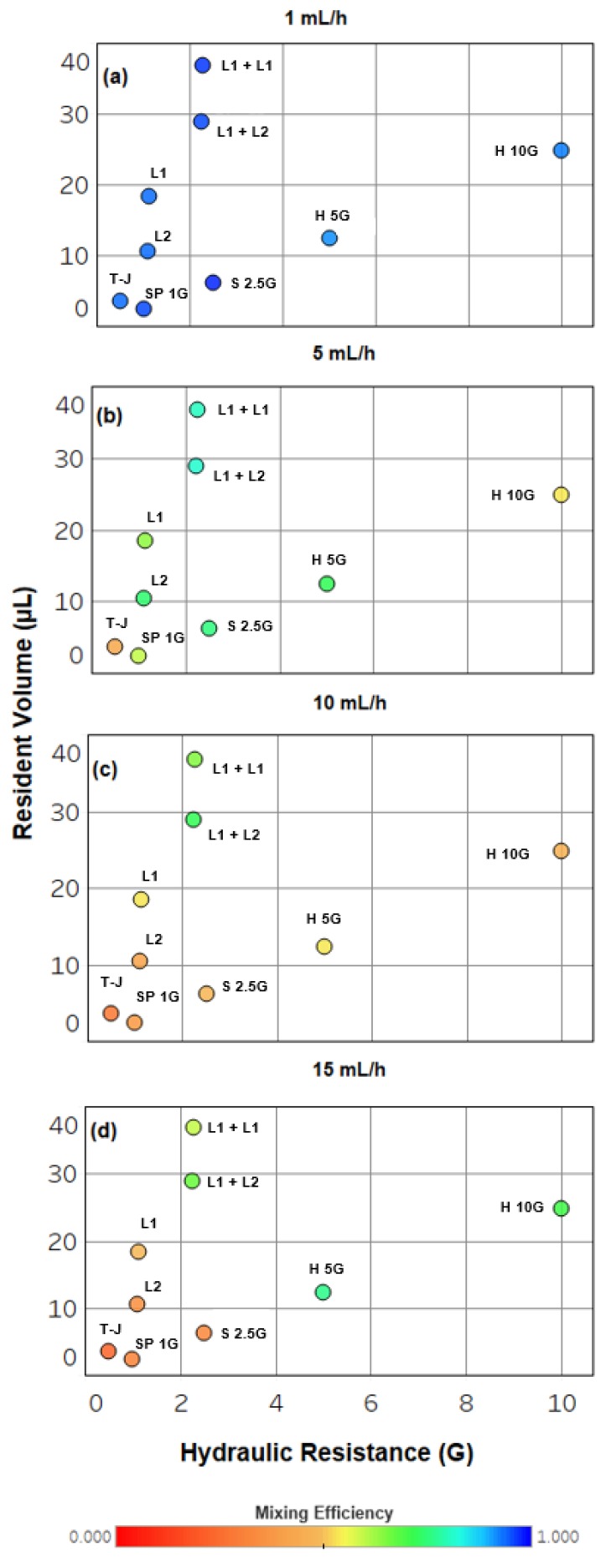
Trade-off between mixing efficiency, hydraulic resistance, and resident volume for all devices and configurations studied at four flow rates: (**a**) 1 mL/h, (**b**) 5 mL/h, (**c**) 10 mL/h, and (**d**) 15 mL/h. The legend corresponds to mixing efficiency where red markers have the poorest mixing and blue markers have the best mixing. The relative positions of points representing each component are as labeled in (**a**).

Figure 4 represents a map of the design trade-off between resident volume, hydraulic resistance, and mixing efficiency over a broad range of flow rates for the device configurations studied. At low flow rates, roughly <5 mL/h, all devices studied here achieve similar mixing efficiencies, enabling designers with flexible choice of components based on resistance and resident volume characteristics. More specifically, the H 5G device accomplishes nearly equivalent mixing efficiencies as the series laminator configurations (L1 + L1 and L2 + L2) with far less cost to resident volume. The H 10G device behaves much like the individual L1 and L2 devices with only slightly worse cost to resident volume. At high flow rates (>15 mL/h), helical devices such as H 5G and H 10G show similar, advantageous mixing efficiencies relative to others, but the H 5G device outperforms the H 10G with respect to conserving resident volume budget. The laminator devices appear to best serve designers with high mixing efficiency at low to moderate flow rates (<10 mL/h) where minimal impact on network resistance is desired. This is at the expense of slightly higher volumes relative to helical devices. For microfluidic networks operated typically at low flow rates, designers have good access to a variety of resistors in choosing helical devices. However, laminator devices will ensure less sensitivity over a wider range of flow rates, acting to functionally stabilize mixing in the network to unintended operational defects. A common example of this is the opening and closing of downstream valves, which can cause rapid pulses in fluid pressure, and therefore flow rate.

## 3. Materials and Methods

### 3.1. Microfluidic Experiments

The discrete microfluidic elements used in this study were manufactured using stereolithography from DSM Somos Watershed XC 11122 photoresin in the manner previously reported [13,14,15,16,18]. Microfluidic channels for all components were designed with 642.5 μm × 642.5 μm square cross sections, in keeping with the previously established standards [13]. Test assemblies as shown in Figure 2 were assembled by hand, with special care taken to correctly orient L1 devices such that inlet streams were correctly split and recombined to enhance mixing as described in Section 2.1. Assemblies were observed using a Leica M80 stereoscope (Leica Microsystems Inc., Buffalo Grove, IL, USA) with attached Leica DFC 310 FX camera (Leica Microsystems Inc.). Eight-bit monochromatic TIFF files were recorded over a range of flow rates. In each image collected, the same illumination and camera exposure settings were used, with attention paid to maintaining unity gamma such that intensity is linear with concentration of dye for any sample. Flows of dye and water into experimental assemblies were managed using a KDS Legato 210P push-pull style syringe pump (KD Scientific Inc., Holliston, MA, USA) and 1/16” OD PEEK (green) tubing. The 2 mg/mL Coomassie Blue in deionized (DI) water was used for the dyed streams for collecting all data presented in Figure 3 and Figure 4. Generic food dyes diluted into DI water were used in visualization experiments, such as in Figure 1.

### 3.2. Data Analysis

All data processing was performed using the R programming language in the RStudio developer environment. As indicated in Figure 2, the intensity along a line was measured perpendicular to the channel walls in a SP 1G element in series with the mixer element or configuration of elements being measured. An intensity profile was constructed by locating the centre of this line, and then storing values associated within 50 pixels to the right and left of the centre. The edges of the channel walls themselves were found by first measuring the mean intensity value and standard deviation of intensity in 100 pixels at the outer edges of the profile, and then searching for a change in intensity greater than three standard deviations from the left and right edges of the profile to the centre, marking the approximate locations of the edges. The interior of the channel was then determined within a margin of eight pixels of the approximate edges to exclude noise due to the channel–fluid interface. Data for the mixed condition used to baseline illumination inhomogeneity was collected at a flow rate of 0.25 mL/h through the centre of the experimental assembly (Figure 2).

## 4. Conclusions

We have successfully demonstrated a new class of 3D-printed, discrete microfluidic elements for high-efficiency mixing over a variety of flow rates. Additive manufacturing was used to fabricate devices achieving intricacy not made possible by standard manufacturing methods (e.g., micromachining). To experimentally quantify the performance of mixing elements, a new method of assessing mixing efficiency was developed. With this, we show the trade-off between the resident volume, hydraulic resistance, and the mixing efficiency achievable with these elements. As such, users with knowledge of flow rates and available reagent volumes acceptable to their experiments can choose components from the standardized library that will adequately satisfy mixing efficiencies and reagent volume.

## Supplementary Materials

The following are available online at www.mdpi.com/2072-666X/8/5/137/s1, Table S1: Standard library of components used in these experiments, which include: mixers, straight pass, port, and connectors. Here we include a CAD image of the devices, their respective nomenclature (e.g., L1, L2, R1G, etc.), their network resistance model, and lastly the devices respective resistance and associated resident volume; Figure S1: Alternate views of the L1 laminator discrete element showing a side-view (a), top-down view (b) and an interior view (c). Likewise, the bottom row of images shows alternate views of the L2 laminator discrete element showing a rear-view (d), side-view (e), and an interior view (f).; Figure S2: Process flow for determining mixing efficiency of a device. The process begins by using a stereoscope to take images of the system in question at different flow rates. The pixel intensity for a line perpendicular to the center of the straight pass channel in the system is isolated. This is done for a total of 101 intensity profiles, by taking profiles to the left and right of channel center point, 50 pixels in range, respectively. This data is averaged and put through a flatness correction to adjust for uneven lighting that may occur. Data is then normalized and mixing efficiency is determined.; Figure S3: A linear fit around the measured line intensity of a ‘perfectly mixed’ situation run at 0.5 mL/h was used to correct for uneven illumination of other trials at higher flow rates. The fitted line was subtracted from measured intensity of relevant flow rates. An example case is shown here for the measured intensity perpendicular to the center of a channel for the 1L device at three different flow rates: 0.5, 10, and 20 mL/h. Flattened data is later normalized to determine mixing efficiency.
