# Supplementary material for: Modular, Discrete Micromixer Elements Fabricated by 3D Printing
Source: Micromachines (Basel). 2017 Apr 26;8(5):137. doi: 10.3390/mi8050137 (PMC6190369; doi:10.3390/mi8050137)
Supplement: Supplementary file 1 [file micromachines-08-00137-s001.pdf]

# Supplementary Materials: Modular, Discrete Micromixer Elements Fabricated by 3D Printing

**Krisna Bhargava, Roya Ermagan, Bryant Thompson, Andrew Friedman and Noah Malmstadt**

**Table S1.** Standard library of components used in these experiments, which include: mixers, straight pass, port, and connectors. Here we include a CAD image of the devices, their respective nomenclature (e.g. L1, L2, R1G, etc.), their network resistance model, and lastly the devices respective resistance and associated resident volume.

| Class | Image | Nomenclature | Network | Resistance (G) | Resident Volume (μL) |
|---|---|---|---|---|---|
| **Mixer** | 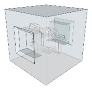 | L1 1.133G | 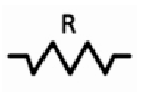  | 1.130 | 18.396 |
| | 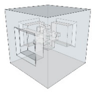 | L2 0.768G<br>0.339G | 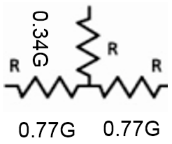  | 0.770<br>0.340 | 10.487 |
| | 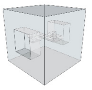 | S 2.5G | 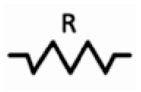  | 2.5 | 6.192 |
| | 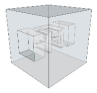 | H 5G | 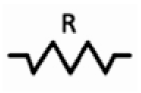  | 5 | 12.384 |
| | 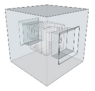 | H 10G | 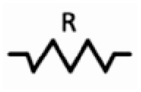  | 10 | 24.768 |
| **Junction** | 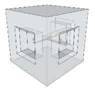 | T 0.5G | 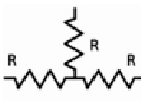  | 0.5 | 3.582 |
| **Straight Pass** | 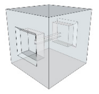 | SP 1G | 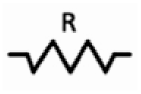  | 1 | 2.476 |
| **Port** | 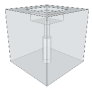 | P 0.5G | 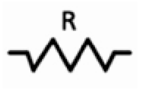  | 0.5 | 1.238 |
| **Connector** | 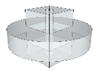 | C 1G | 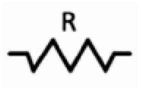  | 1 | 2.476 |

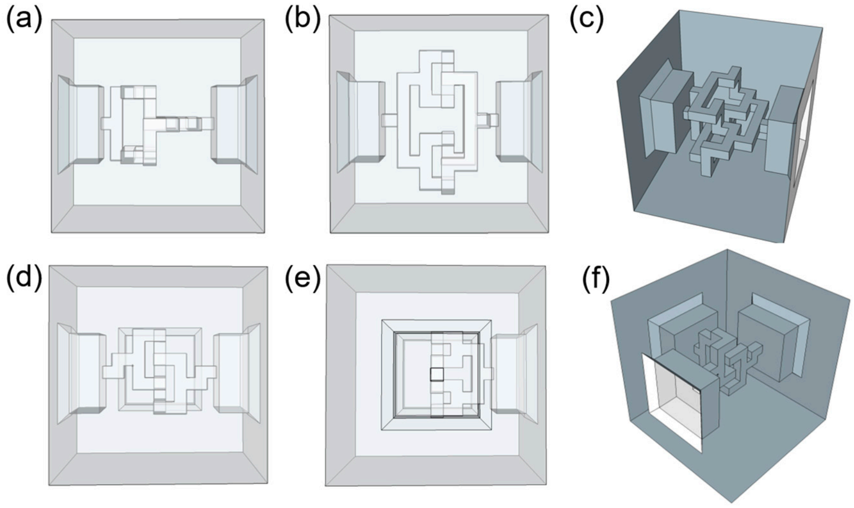


**Figure S1.** Alternate views of the L1 laminator discrete element showing a side-view (a), top-down view (b) and an interior view (c). Likewise, the bottom row of images shows alternate views of the L2 laminator discrete element showing a rear-view (d), side-view (e), and an interior view (f).

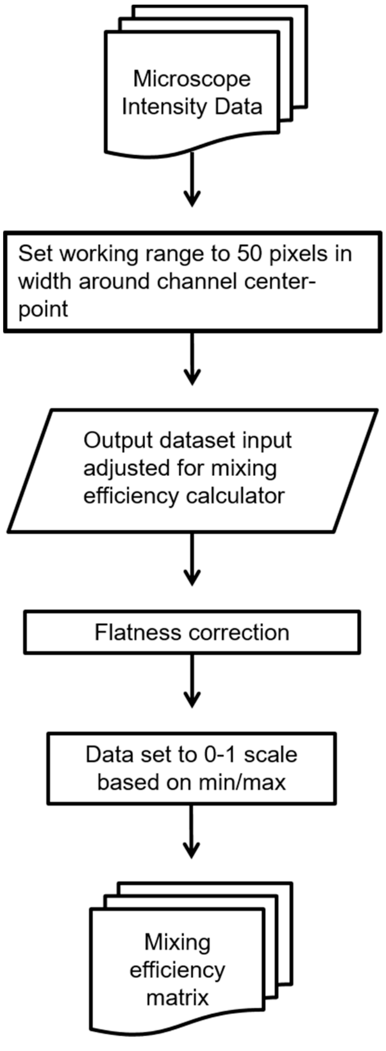


**Figure S2.** Process flow for determining mixing efficiency of a device. The process begins by using a stereoscope to take images of the system in question at different flow rates. The pixel intensity for a line perpendicular to the center of the straight pass channel in the system is isolated. This is done for

a total of 101 intensity profiles, by taking profiles to the left and right of channel center point, 50 pixels in range, respectively. This data is averaged and put through a flatness correction to adjust for uneven lighting that may occur. Data is then normalized and mixing efficiency is determined.

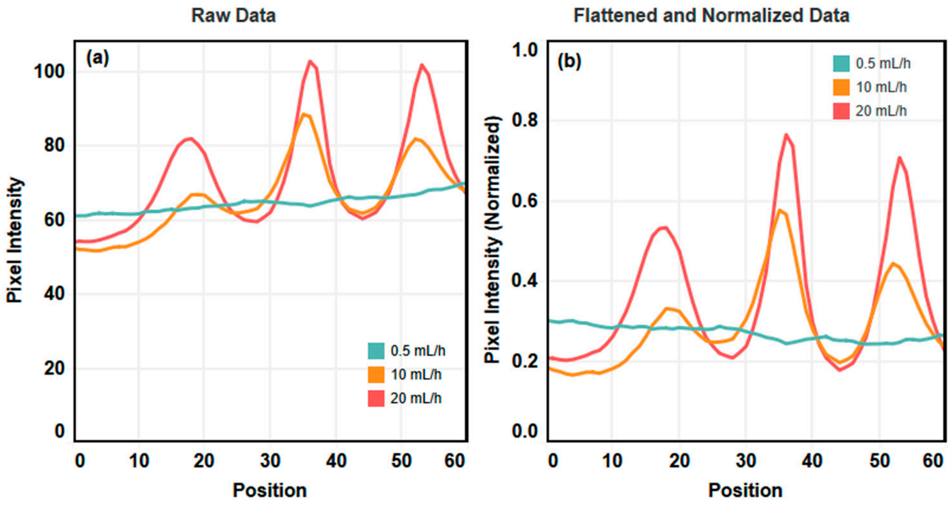


**Figure S3.** A linear fit around the measured line intensity of a 'perfectly mixed' situation run at 0.5 mL/h was used to correct for uneven illumination of other trials at higher flow rates. The fitted line was subtracted from measured intensity of relevant flow rates. An example case is shown here for the measured intensity perpendicular to the center of a channel for the 1L device at three different flow rates: 0.5, 10, and 20 mL/h. Flattened data is later normalized to determine mixing efficiency.
